# Biomechanical Determinants of Anterior Cruciate Ligament Stress in Individuals Post–ACL Reconstruction During Side-Cutting Movements

**DOI:** 10.3390/bioengineering12030222

**Published:** 2025-02-22

**Authors:** Huijuan Shi, Yuanyuan Yu, Hongshi Huang, Hanjun Li, Shuang Ren, Yingfang Ao

**Affiliations:** 1 Key Laboratory for Performance Training & Recovery of General Administration of Sport, College of Human Movement Science, Beijing Sport University, Beijing 100084, China; shihuijuan1103@163.com (H.S.); lihanjun@bsu.edu.cn (H.L.); 2 Department of Sports Medicine, Peking University Third Hospital, Institute of Sports Medicine of Peking University, Beijing 100191, China

**Keywords:** ACL reconstruction, side-cutting, risk factor, biomechanics

## Abstract

This cross-sectional laboratory-based study investigates the stress characteristics of the anterior cruciate ligament (ACL) during side-cutting using a knee finite element (FE) model and identifies biomechanical factors influencing ACL stress. Kinematics and ground reaction forces (GRF) were collected from eight participants (age: 30.3 ± 5.3 years; BMI: 25.6 ± 2.4 kg/m^2^; time since surgery: 12.8 ± 1.2 months) one year post–ACL reconstruction during side-cutting tasks. A knee FE model incorporating time-varying knee angles, knee forces, and femoral translation was developed to simulate the knee biomechanics. The relationships between ACL stress and lower limb biomechanics were analyzed. The results indicated the highest stress concentrations at the femoral attachment during the early landing phase. Posterior femoral displacement relative to the tibia was significantly correlated with peak ACL equivalent stress (r = 0.89, *p* = 0.003) and peak ACL shear stress (r = 0.82, *p* = 0.023). Peak ACL equivalent stress also showed positive correlations with posterior GRF (r = 0.77, *p* = 0.025) and knee extension moments (r = 0.71, *p* = 0.049). In contrast, peak ACL shear stress exhibited a significant negative correlation with hip extension moment (r = −0.80, *p* = 0.032). This study identified key biomechanical factors affecting ACL stress, highlighting the roles of femoral displacement, knee extension moments, and ground reaction forces, while demonstrating a negative relationship with hip extension moments.

## 1. Introduction

Anterior cruciate ligament (ACL) injury is one of the most common sports injuries. Anterior cruciate ligament reconstruction (ACLR) has become the standard procedure to restore knee stability [1]. However, studies reported that the risk of reinjury to the reconstructed ACL or injury to the contralateral ACL remains high post-ACLR [2,3]. In addition, individuals after an ACL reconstruction had a high prevalence of osteoarthritis [4]. Thus, it is essential to identify the risk factors associated with ACL injuries to reduce the re-injury rate and improve the rehabilitation outcome after ACLR.

A wide array of studies have delved into the risk factors of ACL injuries, employing diverse methodologies that may contribute to the variability in the findings [5]. While cadaveric experiments can directly measure ligament strains under controlled conditions [6], they often fail to capture the dynamic stresses experienced during actual sports activities. Although video analysis has helped clarify movement patterns, it falls short in quantifying ACL loads [7,8]. Recent studies have used a high-speed fluoroscopic system to analyze ACL length changes and strains during activities such as walking and jumping [9,10]. However, the limited testing space of these systems makes it challenging to assess high-risk maneuvers like side-cutting that demand an extensive range of motion. Beyond those experimental studies, finite element (FE) modeling has been used to analyze the ACL stresses that are difficult to measure directly [11,12,13,14].

Finite element modeling has been extensively utilized to explore biomechanical risk factors associated with ACL injuries. Orsi et al. [13] found that at a 25° knee flexion angle, the combination of knee valgus and internal femoral rotation substantially heightens ACL stress. Similarly, Homyk et al. [14] investigated the effect of femoral rotation and valgus moments at the same knee flexion angle, discovering that valgus moments posed a higher risk for ACL injuries compared to femoral rotation. Further investigation into ACL stress at varying knee flexion angles (0°, 10°, 20°, 30°, and 45°) revealed that the highest stress and strain on the ACL occurred at full extension (0°) [15]. Although previous studies have explored ligament stress under different loading conditions [13,16], they have not sufficiently addressed dynamic movements such as side-cutting, which is well recognized for its high risk of ACL rupture [17]. Dynamic modeling of athletic movements offers a valuable approach for assessing how multi-planar loading affects ACL biomechanics, shedding light on the risk factors during complex sports activities. By integrating experimental measurements with computational models, we can gain a more comprehensive understanding of ACL loading patterns during high-risk dynamic tasks, potentially advancing injury prevention strategies.

Given that the re-rupture rates for both graft and contralateral ACL post-ACLR are nearly the same [3], this population likely retains risk factors for ACL injury, making them more susceptible than the general population. Therefore, this study aims to analyze ACL loading characteristics during side-cutting in individuals post-ACLR and identify movement characteristics linked to ACL loading. We hypothesize that the biomechanical parameters in the sagittal plane of the knee and hip joints during side-cutting would affect ACL stress. We further hypothesize that frontal plane movement characteristics of the knee and hip joints would be associated with ACL stress.

## 2. Materials and Methods

### 2.1. Participants

This cross-sectional laboratory-based study aimed to investigate biomechanical determinants of ACL stress during side-cutting movements in individuals post-ACLR. Eight male patients were recruited from Peking University Third Hospital (Beijing, China) between January 2021 and June 2021 for this study. The sample size of the eight participants was chosen based on the exploratory nature of this study. All the participants were recreationally active and one year post–ACL reconstruction (age: 30.3 ± 5.3 years; body height: 1.76 ± 0.05 m; body weight: 79.6 ± 8.6 kg; BMI: 25.6 ± 2.4 kg/m^2^; time since surgery: 12.8 ± 1.2 months). They had undergone autologous hamstring tendon reconstruction with standard rehabilitation protocols [18] and had no prior lower limb surgeries beyond ACL reconstruction. The exclusion of female participants was intentional to avoid confounding effects due to known sex differences in ACL injury rates and biomechanics. Future studies should consider including both male and female participants to assess potential sex-related differences in ACL stress and biomechanics.

All the participants were one year post-ACLR. All the participants had undergone autologous hamstring tendon reconstruction with standard rehabilitation protocols without additional surgeries on other structures and had no prior surgical interventions on the lower limbs. All the participants went through regular postoperative home-based rehabilitation programs. The study protocol was approved by the ethics committee of the hospital (IRB00006761-M2019056) on 5 March 2020, and all the participants provided written informed consent before data collection.

### 2.2. Data Collection and Analysis of Side-Cutting Movement

Marker-based motion capture was used to measure kinematics. Passive reflective markers were placed bilaterally on key anatomical landmarks: the anterior superior iliac spine, posterior superior iliac spine, lateral thigh, lateral femoral condyle, medial femoral condyle, lateral tibial plateau, medial tibial plateau, anterior superior shank, anterior inferior shank, lateral malleoli, medial malleoli, heel, and first and fifth metatarsophalangeal. A static calibration file in the standing position was recorded for each participant before the movement test. In a side-cutting test, the participant was instructed to complete the movement as quickly as they were comfortable with. Each participant completed three successful trials, defined as those where the participant executed the movement according to the protocol, allowing for comprehensive kinematic and kinetic data collection. Three-dimensional (3D) trajectories of the reflective markers were collected using an eight-camera motion capture system (VICON, Oxford, UK) at a sample rate of 100 Hz. Ground-reaction forces (GRF) were collected using two embedded force plates (AMTI, Watertown, MA, USA) at a sample rate of 1000 Hz.

To address the discrepancy between the sampling frequencies of the motion capture system (100 Hz) and the force plate (1000 Hz), we increased the sampling frequency of the kinematic data to 1000 Hz using Visual 3D software. This adjustment allowed for proper alignment of the motion capture data with the force plate data, enabling accurate dynamic calculations. The raw trajectories of the reflective markers were filtered through a Butterworth low-pass digital filter at an estimated optional cutoff frequency of 10 Hz [19]. Kinematic and kinetic variables were calculated using Visual 3D software (C-Motion Inc., Germantown, MD, USA). The parameters analyzed in this study included three-dimensional tibial displacements relative to the femur, knee joint angles, and knee forces, evaluated from the moment of landing to the point of peak posterior GRF.

For precise anatomical referencing, the midpoint between the medial and lateral femoral condyles was aligned to the tibial link coordinate system in both static and dynamic phases of movement. The midpoint between the medial and lateral femoral condyles was transferred to the tibial link coordinate system. The coordinates of the center of the medial and lateral femoral condyles under the tibial coordinate system were calculated in both the static calibration file and the cutting movement file. The relative displacement between the tibia and femur during side-cutting was calculated as the difference between the position of the femur in the dynamic and static files under the tibial coordinate system. In addition, to correct potential scaling errors due to the markers, the distance between the medial and lateral condyles of the femur in the coronal plane was measured from MRI images. The ratio was determined between the distances of the medial and lateral femoral condyles in the MRI images and those derived from reflective markers in the collected static files. Finally, this ratio was used as a scaling factor to adjust the relative displacement of the femur and tibia, thus ensuring model accuracy.

### 2.3. Finite Element Analysis

The stress characteristics of the ACL during side-cutting were analyzed using a finite element model of the knee joint, as previously described in the literature [16]. The material properties of the knee model were derived from literature values [20,21,22,23]. The model included a designated femoral reference point located at the midpoint between the lateral and medial femoral condyles. A kinematic coupling relationship was established between this reference point and the corresponding femoral node to ensure accurate transmission of movement and force within the model. In the simulation protocol, the degrees of freedom for both the tibia and fibula were constrained to mimic the physiological constraints present during dynamic activities. The loading conditions applied to the femoral reference point included knee joint angles, relative translations of the femur with respect to the tibia, and the net forces acting on the knee joint. These simulations were conducted using ABAQUS/Explicit software, focusing specifically on the critical phase from the moment of landing to the moment of peak posterior GRF. During this interval, the ACL stress was calculated to assess its response to the applied loads.

**Figure 1:**
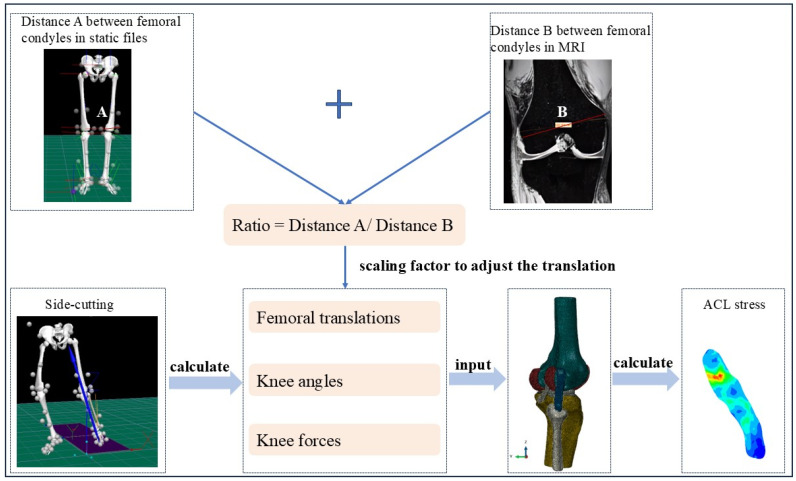
Data collection and analysis process. In the ACL stress distribution map, the color gradient indicates relative stress magnitude, with red representing the highest stress, followed by yellow, green, and blue (lowest stress).

The key input variables for the FE simulation were the displacement of the tibia relative to the femur, the 3D knee joint angles, and the 3D force parameters, all of which were loaded on the femur reference point. The knee joint in the FE model was assumed to start from a neutral, fully extended position (straight knee), and the applied boundary conditions simulate dynamic loading during side-cutting. Boundary conditions for the model were defined by the time-varying knee angles and tibial translations, reflecting the real-time kinematics of the knee during side-cutting. Additionally, the time-varying knee forces, representing the dynamic loading conditions, were applied throughout the simulated movement (Figure 1).

### 2.4. Data Analysis

At the moment of peak ACL equivalent stress during side-cutting, the biomechanical parameters, including knee angles, knee moments, the relative displacement of the tibia and femur, hip angles, and hip moments, were extracted. The Shapiro–Wilk test was applied to assess the normality of the parameters. The data are presented as mean ± standard deviation for normally distributed data. The Pearson’s correlation coefficient was applied to investigate the relationship between ACL stress and the above biomechanical parameters. The statistical analyses were performed using SPSS version 16.0, with the *p*-value threshold set at 0.05.

## 3. Results

### 3.1. Anterior Cruciate Ligament Stress Characteristics

**Figure 2:**
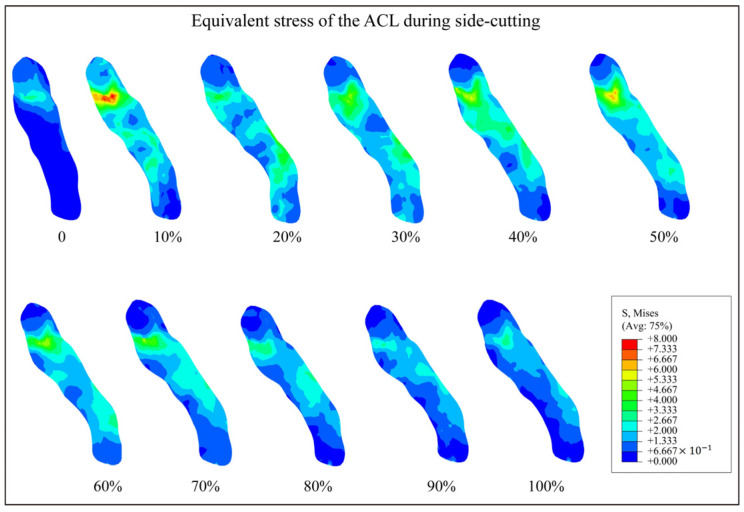
Equivalent stress of the ACL during side-cutting. 0 to 100% indicates the corresponding time phase of the side-cutting. The color scale indicates the magnitude of stress, with blue representing low stress and red representing high stress. Low-stress condition: stress concentrations are observed primarily at the tibial attachment. High-stress condition: significant stress concentrations at the femoral attachment during the early landing phase.

The kinematic and kinetic parameters measured during side-cutting movements were input into the model as loading conditions, and the equivalent stress characteristics of the ACL during side-cutting were calculated, as shown in Figure 2. The results showed that the upper part of the ACL has relatively high stress compared to other areas. The femoral attachment region of the ACL had greater stress than the tibial attachment area, and the stress was greater during the early phases of landing.

**Figure 3:**
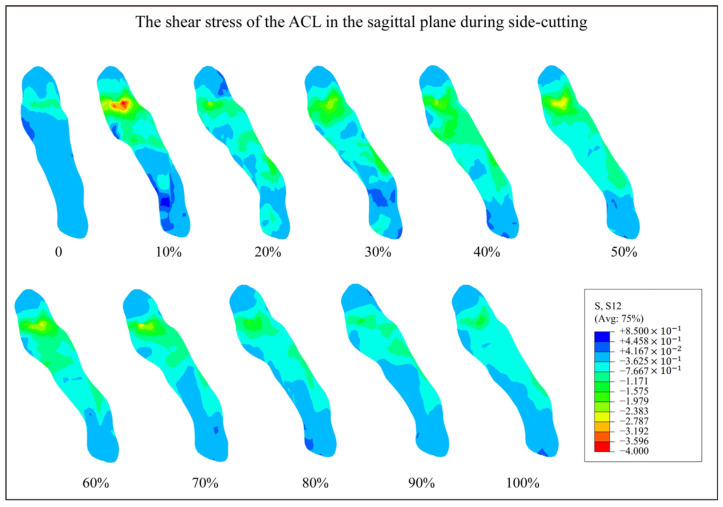
The shear stress of the ACL in the sagittal plane during side-cutting. 0 to 100% indicates the corresponding time phase of the side-cutting. The color scale indicates the magnitude of stress, with blue representing low stress and red representing high stress. Low-stress condition: stress concentrations are observed primarily at the tibial attachment. High-stress condition: significant stress concentrations at the femoral attachment during the early landing phase.

The distribution of shear stress of the ACL in the sagittal plane is shown in Figure 3. The ACL had the highest shear stress initially upon landing, diminishing in intensity through subsequent stages of the movement. Among the various regions of the ACL, the femoral attachment area was most affected by shear stress, followed by the mid-substance of the ligament. Conversely, the tibial attachment area experienced the least amount of shear stress.

### 3.2. Analysis of Biomechanical Factors Affecting ACL Stress

The variables examined in this study were confirmed to be normally distributed. The results of the correlation analysis indicated significant relationships involving the biomechanical dynamics of the knee. Specifically, both posterior and inferior displacements of the femur relative to the tibia were significantly correlated with peak ACL equivalent stress (*p* = 0.003, *p* = 0.034, respectively) (Table 1). Furthermore, ACL stress during side-cutting was found to be positively correlated with posterior GRF and knee extension moments at peak ACL equivalent stress (*p* = 0.025 and *p* = 0.049, respectively). However, no significant correlations were observed between ACL stress and the other variables examined (*p*-values ranging from 0.069 to 0.964).

In terms of ACL shear stress in the sagittal plane, significant correlations were identified. The lateral and posterior displacements of the femur relative to the tibia were significantly correlated with peak ACL shear stress (*p* = 0.049, *p* = 0.023) (Table 2). The results also showed a significant negative correlation between peak ACL shear stress and hip extension moment at peak ACL shear stress during side-cutting (*p* = 0.032). No significant correlations were found between ACL shear stress and the remaining variables analyzed (*p*-values ranging from 0.133 to 0.965).

## 4. Discussion

The findings of this study provided significant insights into the biomechanical dynamics of the ACL during side-cutting maneuvers, revealing specific stress patterns and their correlations with kinematic and kinetic factors. The results highlight the differential stress distributions within the ACL during dynamic side-cutting activities, emphasizing areas of potential vulnerability to injury. This study also underscores the importance of focusing on specific kinematic and kinetic factors, such as femoral displacement, posterior GRF, knee extension moments, and hip extension moments, which have proven to be significant determinants of ACL stress. The study highlights the potential intervention points for training modifications to reduce ACL injury risks, providing a foundation for further research into preventive and rehabilitative strategies.

The results showed that the ACL had significantly higher stress in the upper part of the ligament during side-cutting, with greater stress observed at the femoral attachment compared to the tibial attachment. This finding was consistent with clinical observations, which commonly identify the femoral insertion point as the predominant site of ACL ruptures. Consistent with previous research [24], acute ACL ruptures predominantly occur in the upper part of the ligament, reported at a rate of 72.2%, followed by 26.4% in the middle segment, and are infrequent in the lower segment. These stress characteristics across different segments of the ligament may be influenced by their anatomical configurations. The anatomical structure of the ACL contributes significantly to its strength and susceptibility to injury. The tibial insertion of the ACL, extending forward along the intercondylar ridge of the tibia, features a flat and elongated attachment area. This larger tibial attachment area relative to the femoral insertion likely contributes to enhanced ligament strength, making the tibial insertion less prone to injuries. The results of this study highlight the importance of accurately replicating the structural form of the ligament in biomechanical models to facilitate realistic stress analyses. Moreover, the study revealed that ACL stress peaks during the initial landing phase, specifically within the first 10% of the movement. This highlights a critical window where the risk of ACL injury is markedly elevated, underscoring the need for targeted interventions during this phase to mitigate injury risks.

The correlation analysis results showed that the equivalent stress on the ACL during side-cutting was significantly correlated with the posterior and inferior displacement of the femur relative to the tibia. This posterior displacement arises primarily from anterior shear forces exerted on the tibia. Since the primary physiological function of the ACL is to restrict the anterior translation of the tibia, the anterior displacement relative to the femur places strain on the ACL and increases the forces exerted on the ACL. Additionally, large axial loads are often associated with substantial downward displacement of the femur during movement. Meyer and Haut used cadaveric specimens to simulate jump landing. They found that a large axial impact load on the knee during landing may lead to ACL rupture, typically accompanied by anterior and lateral displacements of the tibia relative to the femur [25]. Schmitz et al. observed that under conditions of near extension or minimal flexion of the knee, increased axial loads could augment the anterior displacement of the tibia [26]. Previous injury prevention training has emphasized the importance of the landing movement pattern, highlighting that a softer landing with an increased knee flexion angle could diminish the impact force. Combined with previous studies, this study further suggests that the higher impact load on the knee joint contributes to heightened ACL stress. Reducing the impact force could, therefore, reduce ACL stress, providing a theoretical basis for developing ACL injury prevention strategies.

The results of the study support the first hypothesis, which suggests that ACL equivalent stress during side-cutting is positively correlated with knee extension moments and posterior GRF. A previous study reported that the asymmetries in vertical and posterior GRF during side-cutting effectively predicted knee extension moment asymmetry [27]. The consistency between the effects of posterior GRF and knee extension moment on ACL forces in this study was consistent with the literature. It has been shown that the increased posterior GRF during the stop-jump movement result in greater quadriceps contraction and greater ACL loading [28]. It has been suggested that posterior GRF and knee extension moments were significantly associated with peak anterior shear forces at the proximal tibia during the stop-jump movement, underscoring their critical role in influencing ACL forces [29]. Previous literature also reported that landing with a greater internal knee extension moment was significantly associated with an increased risk of ACL injury [30]. The internal knee extension moment, driven by quadriceps muscle contraction, especially at smaller knee flexion angles, exerts a force that advances the tibia forward, thereby augmenting the force on the ACL. The inter-limb GRF asymmetries are significant in individuals post-ACLR and may influence ACL loading patterns. Future studies should explore these asymmetries in greater detail to better understand their role in ACL stress and rehabilitation.

A significant negative correlation was found between peak shear stress on the ACL and the hip extension moment, indicating that increased hip extension moments may help reduce the anterior shear stress in the sagittal plane. Typically, hip extension moments are produced by the contraction of the hamstring muscles, which play a crucial role in stabilizing the knee by limiting anterior tibial translation [31]. Shimokochi et al. [32] showed that an increase in hip extension moments was significantly associated with reduced knee extension moments during single-leg landing. They proposed that a forward tilt of the trunk could strategically decrease quadriceps contraction demands while increasing those for hamstring contraction. Further supporting this, Yang et al. [33] developed an injury prevention program that focused on improving landing techniques through targeted training of the hip extension muscles combined with rapid lower limb muscle stretch training. Their findings from a 4-week intervention showed effective reductions in risk factors associated with lower limb injuries during landing movements. The results of this study, combined with previous studies, further suggest that enhancing the contraction of the hamstring during high-risk sports is vital for maintaining stability at the knee and protecting the ACL. Therefore, strength training for the hip extensor muscle group should be a focal point in ACL injury prevention and rehabilitation programs.

This study found that ACL stress is correlated with biomechanical characteristics in the sagittal plane of the knee and hip joints, while no significant correlation was observed with the biomechanical characteristics in the frontal plane. The movement characteristics in patients after ACLR were more uniform in the sagittal plane, whereas variations in the frontal plane tend to be inconsistent and vary considerably across different studies [34]. Movements in the sagittal plane may lead to greater anterior tibial translation or variations in joint angles that place increased stress on the ACL, thereby elevating the risk of injury. On the other hand, the lack of a significant correlation between ACL stress and frontal plane biomechanics implies that varus and valgus motions of the knee and hip may not directly contribute to ACL loading under the conditions studied. This result challenges some common assumptions about the role of knee valgus in ACL injury risk during dynamic tasks [35]. However, it is important to consider that while frontal plane movements may not have a direct impact on ACL stress, they could still indirectly contribute to injury risk through their interaction with sagittal plane motions.

The risk factors during side-cutting movement identified in this study do not entirely align with those identified in previous studies based on bone bruises. Consistent with earlier findings [36,37], this study confirmed that posterior, downward, and medial displacements of the femur relative to the tibia significantly influence ACL forces. These displacements suggest potential joint dislocation during injury and could lead to abnormal loading conditions. In addition, this study found that the primary factors associated with ACL forces during side-cutting include knee and hip extension moments in the sagittal plane and posterior GRF. Earlier research on bone bruises demonstrated that ACL injury mechanisms involve multiple planes. This includes not only the knee flexion state and anterior tibia translation in the sagittal plane but also knee valgus in the frontal plane and tibial rotation in the transverse plane, which are considered high-risk movement patterns that may contribute to ACL injuries [37]. Knee valgus and rotation during side-cutting in this study did not correlate significantly with ACL stress. This discrepancy might indicate that the movement patterns in patients post-ACLR differ from those observed at the initial injury. It is possible that individuals with reconstructed ACL retain a psychological apprehension of re-injury, leading them to subconsciously avoid perceived high-risk movements during physical activities. Patients with ACLR could change their movement control strategies when returning to exercise [38]. The results of this study suggest that the risk factors for reinjury post-ACL reconstruction may differ from those associated with initial ACL injury. Individuals post-ACLR often exhibit altered neuromuscular control [39] and psychological apprehension of re-injury [40]. Future studies should explore the combined effects of biomechanical, neuromuscular, and psychological factors to gain a more comprehensive understanding of ACL injury prevention and rehabilitation.

This study highlights the importance of sagittal plane mechanics in injury prevention and rehabilitation for individuals post-ACLR. The identified biomechanical factors, such as the negative correlation between hip extension moments and ACL shear stress, suggest that hip extensor strength should be prioritized in rehabilitation programs. Neuromuscular training and movement retraining are also essential for optimizing recovery and long-term knee function [41]. While our study focused on biomechanical factors, these findings can inform return-to-sport criteria. Incorporating biomechanical assessments, such as knee and hip extension moments, into decision making could provide a more objective basis for determining an athlete’s readiness to return to high-demand activities. Additionally, recovery strategies play a crucial role in rehabilitation. A systematic review and meta-analysis [42] on pressotherapy for managing muscle soreness after high-intensity tasks, including side-cutting, found moderate benefits but inconclusive effects on overall performance recovery. Therefore, recovery strategies should complement biomechanical assessments to optimize rehabilitation outcomes. Finally, while our study identified associations between biomechanical factors and ACL stress, these do not imply causation or prediction [43]. Future studies are needed to explore the predictive power of these factors for ACL injury or re-injury risk.

The present study focused solely on the biomechanics of side-cutting movements. Further studies are needed to compare ACL stress characteristics across different movement patterns to develop more personalized injury prevention strategies. Another limitation is the potential impact of soft tissue artifacts on kinematics measurements. Although we minimized these artifacts by placing markers on bony landmarks, motion capture systems are inherently susceptible to errors caused by soft tissue movement. Future studies could explore advanced motion capture techniques to reduce soft tissue artifacts and improve data accuracy. The study’s small sample size and male-only sample limit the generalizability of the findings. The exclusion of female participants was intentional to avoid confounding sex differences in ACL injury rates and biomechanics. Future studies should include larger, more diverse samples to assess the impact of sex and other demographic factors on ACL stress and rehabilitation outcomes. Although we observed that hip extension moments were associated with reduced ACL shear stress, we did not analyze other hip and trunk mechanics, which are important for knee stability. Future research should investigate these factors to better understand their role in ACL loading and injury prevention.

## 5. Conclusions

This study investigated the biomechanical behavior of the ACL during side-cutting movements. The results showed that the highest stress concentrations occurred at the upper regions, particularly at the femoral attachment, with peak stress levels observed during the early landing phase. ACL stress was positively correlated with posterior and inferior femoral displacements relative to the tibia, knee extension moments, and posterior ground reaction forces, and negatively correlated with hip extension moments. These findings underscore the importance of sagittal plane mechanics in the context of ACL stress.

## Figures and Tables

**Table 1:** Correlation analysis results between peak ACL equivalent stress and the biomechanical factors at the time of peak ACL equivalent stress.

Variables	Correlation Coefficient	*p* Value
Knee flexion angle	0.48	0.229
Knee abduction angle	−0.13	0.764
Knee external rotation angle	−0.33	0.418
Knee extension moment	0.71	0.049 *
Knee abduction moment	0.14	0.736
Knee external rotation moment	−0.02	0.964
Medial displacement of the femur	0.67	0.069
Posterior displacement of the femur	0.89	0.003 *
Inferior displacement of the femur	0.74	0.034 *
Hip flexion angle	0.15	0.718
Hip extension moment	−0.62	0.099
Hip abduction moment	0.09	0.840
Hip internal rotation moment	0.379	0.354
Posterior ground reaction force	0.77	0.025 *
Vertical ground reaction force	0.50	0.204

* Indicates statistical significance.

**Table 2:** Correlation analysis results between peak ACL shear stress and the biomechanical factors at the time of peak ACL shear stress.

Variables	Correlation Coefficient	*p* Value
Knee flexion angle	0.46	0.305
Knee abduction angle	−0.05	0.916
Knee external rotation angle	−0.37	0.417
Knee extension moment	0.63	0.133
Knee abduction moment	0.15	0.752
Knee external rotation moment	−0.13	0.964
Lateral displacement of the femur	0.76	0.049 *
Posterior displacement of the femur	0.82	0.023 *
Inferior displacement of the femur	0.62	0.135
Hip flexion angle	0.21	0.645
Hip extension moment	−0.80	0.032 *
Hip abduction moment	−0.02	0.965
Hip internal rotation moment	0.36	0.433
Posterior ground reaction force	0.26	0.576
Vertical ground reaction force	−0.21	0.656

* Indicates statistical significance.

## Data Availability

The data presented in this study are available on request from the corresponding author.
